# Supplementary material for: Characteristics of Patients Treated with JAK Inhibitors in Rheumatoid Arthritis before versus after VTE Risk Warnings
Source: J Clin Med. 2022 Dec 27;12(1):207. doi: 10.3390/jcm12010207 (PMC9820905; doi:10.3390/jcm12010207)

Supplementary material

Characteristics of patients treated with JAK inhibitors in rheumatoid arthritis before versus after VTE risk warnings.

Supplemental Table S1. Characteristics of the patients according to treatment, before imputation.

Parameters	Baricitinib<br>n = 155	Tofacitinib<br>n = 77
**Patients baseline characteristics**		
Women	118/155 (76.1)	51/77 (66.2)
Age (years), mean ± SD	59.6 ± 14.2	56.7 ± 13.4
BMI (kg/m²)[1], mean ± SD	27.0 ± 5.9	27.1 ± 6.0
Smoking status		
Non-smoker	62/106 (58.5)	22/57 (46.8)
Former smoker	22/106 (20.8)	12/57 (25.5)
Current smoker	22/106 (20.8)	13/57 (27.7)
**Rheumatoid arthritis characteristics**		
RA duration (years)[2], median (IQR)	11 (4 to 20)	11 (6 to 19)
Seropositivity status		
RF + / ACPA +	122/154 (79.2)	54/76 (71.1)
RF + / ACPA -	11/154 (7.1)	8/76 (10.5)
RF - / ACPA +	8/154 (5.2)	7/76 (9.2)
RF - / ACPA -	13/154 (8.4)	7/76 (9.2)
Erosion presence	101/148 (68.2)	55/73 (75.3)
Extra-articular involvement	28/155 (18.1)	17/77 (22.1)
Surgery related to RA	41/155 (26.5)	19/77 (24.7)
Baseline CRP (mg/L)[3], median (IQR)	7.0 (1.1 to 25.6)	9.9 (2.6 to 27.0)
**Previous treatments**		
Previous Methotrexate	140/155 (90.3)	67/77 (87.0)
Previous TNF inhibitor	117/155 (75.5)	59/77 (76.6)
Previous bDMARD-OMA	107/155 (69.0)	53/77 (68.8)
Prior bDMARD		
Naïve (0)	23/155 (14.8)	7/77 (9.1)
1 or 2	50/155 (32.3)	34/77 (44.2)
3 or more	82/155 (52.9)	36/77 (46.8)
**Comorbidities of special interest**		
Arterial hypertension	48/155 (31.0)	28/77 (36.4)
Diabetes	21/155 (13.5)	13/77 (16.9)
Dyslipidemia	40/155 (25.8)	20/77 (26.0)
Coronary heart disease	11/155 (7.1)	6/77 (7.8)
Peripheral artery disease	7/155 (4.5)	2/77 (2.6)
Stroke	11/155 (7.1)	6/77 (7.8)
Permanent atrial fibrillation	8/155 (5.2)	3/77 (3.9)
Sleep apnea syndrome	14/155 (9.0)	4/77 (5.2)
Pulmonary embolism	7/155 (4.5)	2/77 (2.6)
Deep venous thrombosis	8/155 (5.2)	3/77 (3.9)
Neoplastic history	11/155 (7.1)	6/77 (7.8)
Chronic obstructive pulmonary disease	7/154 (4.5)	6/77 (7.8)
Migraines	1/155 (0.6)	5/77 (6.5)
Herpes zoster infections	7/153 (4.6)	5/76 (6.6)
**Other treatments at baseline**		
Concomitant csDMARD		
None	92/153 (60.1)	47/75 (62.7)
Methotrexate	52/153 (34.0)	22/75 (29.3)
Salazopyrine	0/153 (0.0)	3/75 (4.0)
Leflunomide	7/153 (4.6)	3/75 (4.0)
Hydroxychoroquine (plaquenil)	2/153 (1.3)	0/75 (0.0)
Prednisolone baseline	87/155 (56.1)	32/77 (41.6)
Anticoagulant baseline	6/155 (3.9)	4/77 (5.2)
**Treatment characteristics**		
JAK inhibitor posology		
Classic RA posology	126/155 (81.3)	76/77 (98.7)

Reduced RA posology	29/155 (18.7)	1/77 (1.3)
Introduction before May 2019	107/155 (69.0)	54/77 (70.1)
**Treatment discontinuation**		
Discontinuation	78/155 (50.3)	35/77 (45.5)
Reasons for discontinuation		
Primary inefficacy	24/78 (30.8)	14/35 (40.0)
Secondary inefficacy	24/78 (30.8)	7/35 (20.0)
Adverse events	23/78 (29.4)	11/35 (31.4)
Other (pregnancy, lack of observance, decompensation of comorbidities, no apparent reason)	7/78 (9.0)	3/35 (8.6)

Values are no./total no. (%) unless otherwise stated. [1] 71 missing values (n=52 vs. 19) [2] 6 missing values (n=4 vs. 2) [3] 35 missing values (n=20 vs. 15)

Abbreviations: ACPA = anti-citrullinated protein antibody; bDMARD = biological disease-modifying antirheumatic drug; BMI = body mass index; CRP = C-reactive protein; csDMARD = conventional synthetic disease-modifying antirheumatic drug; IQR = interquartile range; OMA = other mechanism of action; RA = Rheumatoid arhtritis; RF = rheumatoid factor; SD = standard deviation; TNF = tumor necrosis factor.

Supplemental Figure S1. Study Flow chart.

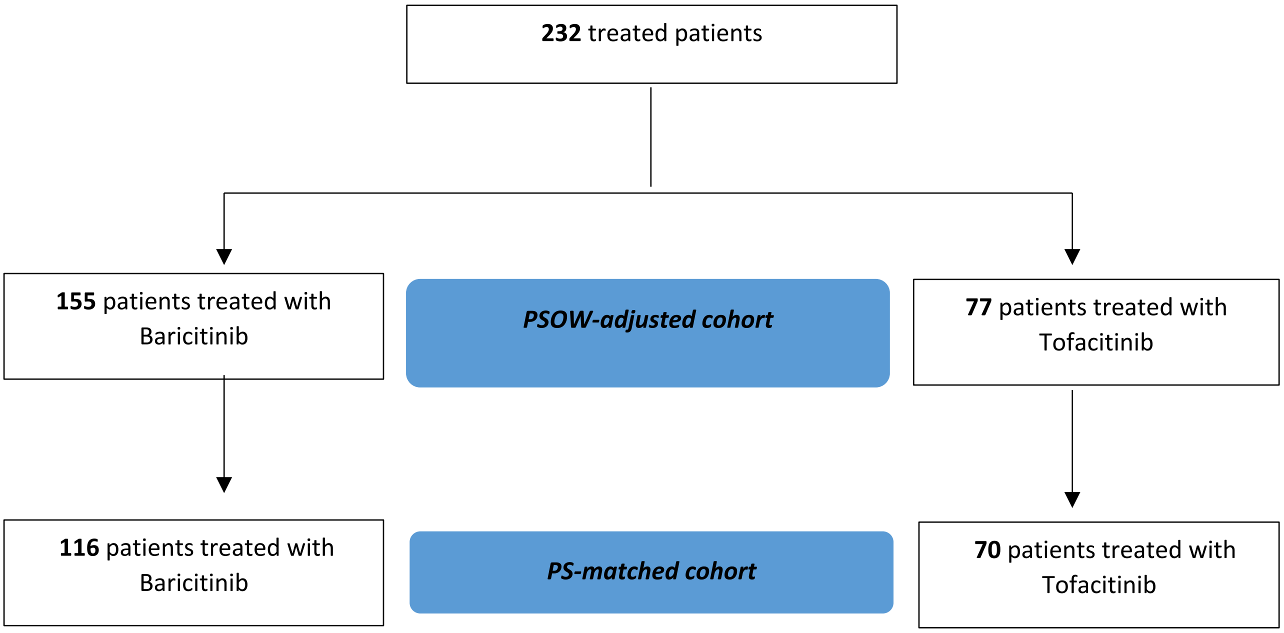

Abbreviations: PS = propensity score; PSOW = propensity score weighting using overlap weights.

Supplemental Figure S2. Two-year follow-up treatment persistence, with vs. without concomitant csDMARD at baseline.

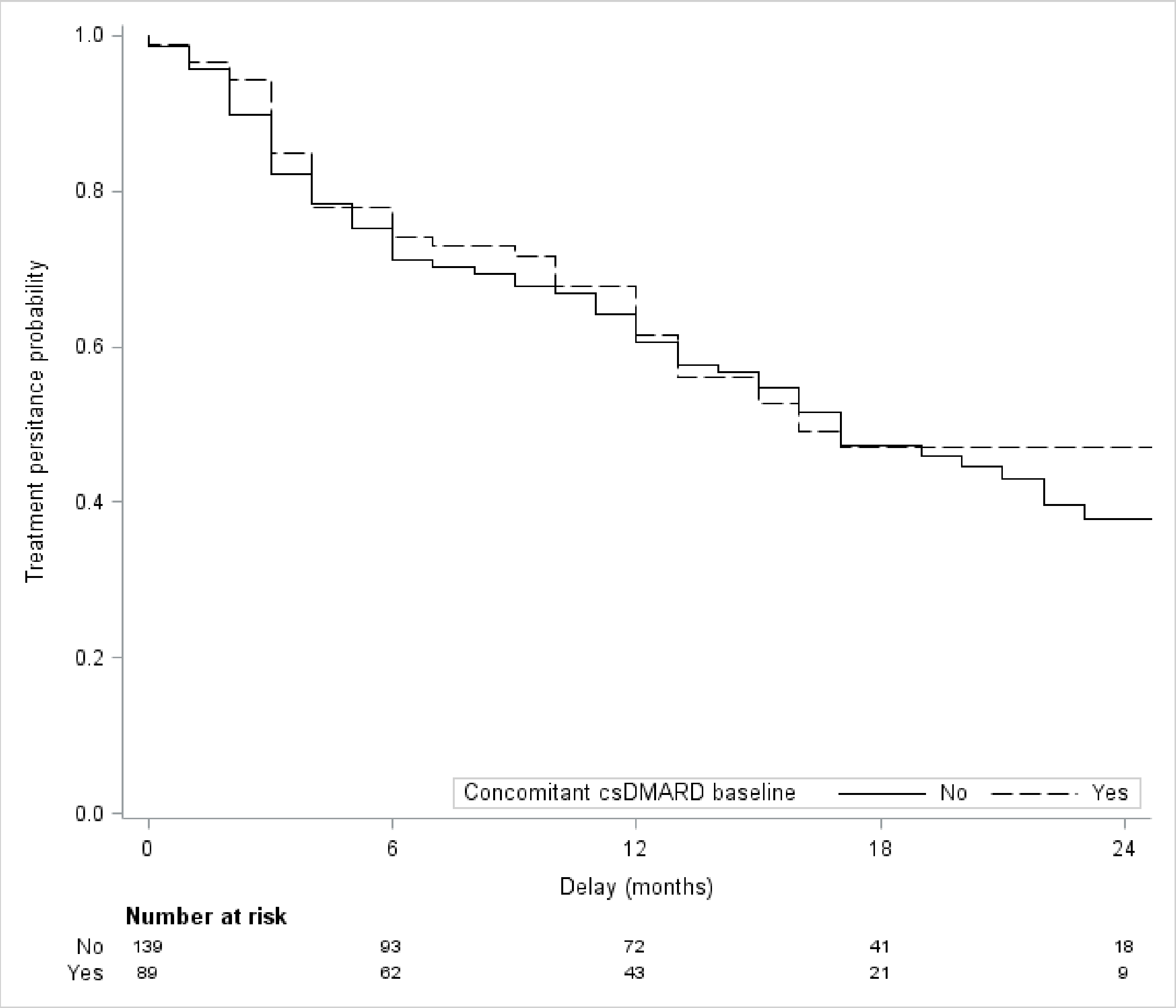

Supplemental Figure S3. Two-year follow-up treatment persistence depending on previous bDMARD status.

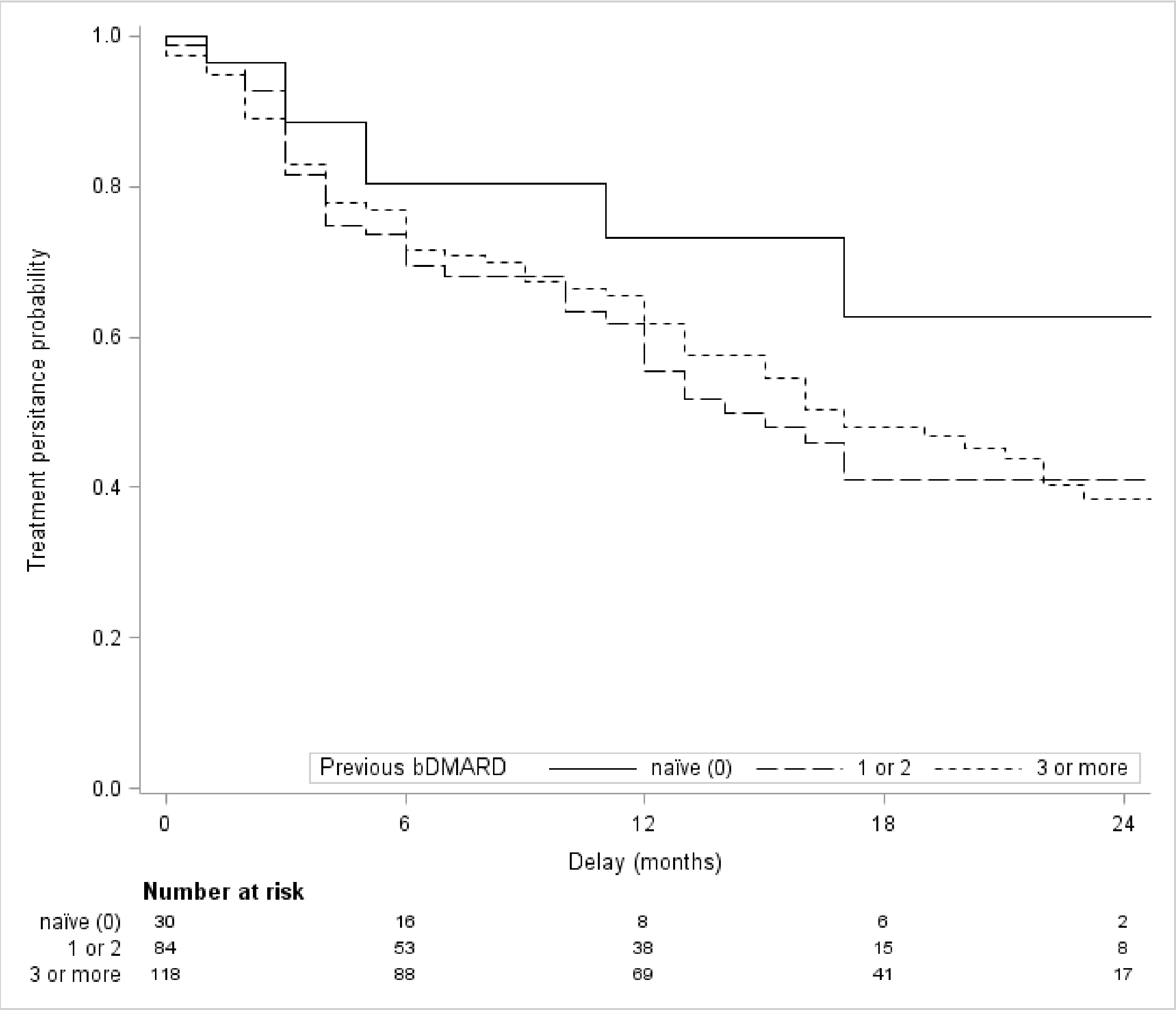

Supplement: Supplementary file 1 [file jcm-12-00207-s001.zip › jcm-2067939-supplementary.pdf]
